# Cullin 4B Ubiquitin Ligase Is Important for Cell Survival and Regulates TGF-β1 Expression in Pleural Mesothelioma

**DOI:** 10.3390/ijms241713410

**Published:** 2023-08-29

**Authors:** Jessica Kreienbühl, Sakunthip Changkhong, Vanessa Orlowski, Michaela B. Kirschner, Isabelle Opitz, Mayura Meerang

**Affiliations:** Department of Thoracic Surgery, University Hospital Zürich, 8091 Zürich, Switzerlandvanessa.orlowski@outlook.com (V.O.); michaela.kirschner@usz.ch (M.B.K.); isabelle.schmitt-opitz@usz.ch (I.O.)

**Keywords:** pleural mesothelioma, cullin 4B, cullin 4A, pevonedistat, TGFβ, MMP2, YAP, CTGF

## Abstract

We previously demonstrated that cullin 4B (CUL4B) upregulation was associated with worse outcomes of pleural mesothelioma (PM) patients, while the overexpression of its paralog CUL4A was not associated with clinical outcomes. Here, we aimed to identify the distinct roles of CUL4B and CUL4A in PM using an siRNA approach in PM cell lines (ACC Meso-1 and Mero82) and primary culture. The knockdown of CUL4B and CUL4A resulted in significantly reduced colony formation, increased cell death, and delayed cell proliferation. Furthermore, similar to the effect of CUL4A knockdown, downregulation of CUL4B led to reduced expression of Hippo pathway genes including YAP1, CTGF, and survivin. Interestingly, CUL4B and not CUL4A knockdown reduced TGF-β1 and MMP2 expression, suggesting a unique association of CUL4B with this pathway. However, the treatment of PM cells with exogenous TGF-β1 following CUL4B knockdown did not rescue PM cell growth. We further analyzed ACC Meso-1 xenograft tumor tissues treated with the cullin inhibitor, pevonedistat, which targets protein neddylation, and observed the downregulation of human TGF-β1 and MMP2. In summary, our data suggest that CUL4B overexpression is important for tumor cell growth and survival and may drive PM aggressiveness via the regulation of TGF-β1 expression and, furthermore, reveal a new mechanism of action of pevonedistat.

## 1. Introduction

Pleural mesothelioma (PM) is an aggressive tumor arising from the mesothelial cell layer lining within the thoracic cavity. Exposure to the carcinogenic mineral asbestos is considered the general cause of PM [1]. Although asbestos usage is banned in a total of 68 countries around the world as of 13 July 2022 (according to www.ibasecretariat.org, accessed on 13 September 2022), the incidence of PM is still rising due to the long disease latency of about 40 years [2]. Moreover, asbestos is still used in many developing countries of the world [3]. In addition, modern materials with a similar structure to asbestos, such as some forms of multiwall carbon nanotubes, may induce asbestos-like disease [4,5].

The treatment of PM is challenging, and the prognosis remains poor, especially because of poor treatment response and tumor recurrence within a median time of 10–18 months after initial treatment [3]. Exploring factors driving aggressive PM phenotypes remains crucial for the identification of new treatments. We previously demonstrated that high expression of cullin 4B (CUL4B), a member of the cullins-RING ligase protein family, was associated with worse outcomes of PM patients [6]. This prompted us to further investigate the mechanistic role of CUL4B in PM.

Cullins form complexes with other proteins and catalyze the ubiquitination of target proteins for proteasomal degradation or activity changes [7]. Two cullin 4 paralogs, CUL4A and CUL4B, share 82% similarity in protein sequence. Both paralogs have been shown to be associated with tumorigenesis and the progression of various tumors [8,9]. In PM, the CUL4A protein levels were elevated in 64% of tumors compared to normal tissues [10], but there was no association between CUL4A and clinical outcomes [6]. In our previous work, we showed that CUL4B was significantly upregulated in PM tumor tissues compared to non-cancerous inflammatory pleural tissues. High expression of CUL4B was associated with short progression-free survival (PFS), and this was further confirmed using the gene expression dataset from TCGA [7].

The CUL4B protein contains an extra nuclear localization signal in the N-terminal sequence, compared to CUL4A [11]. Thus, the majority of CUL4B is localized in the nucleus, whereas CUL4A is mainly localized in the cytoplasm of cells, including mesothelioma tumor cells [6,11]. Thus, it can be speculated that CUL4B has unique functions within the nucleus, which are distinct from CUL4A. Indeed, a nuclear function of CUL4B has been discovered, such as histone modification and epigenetic regulation of various target genes [11]. In neuronal cells, CUL4B ubiquitinates WDR5, a component of the histone methyltransferase complex, for degradation [12]. The depletion of CUL4B caused increased levels of H3K4me3 via WDR5 stabilization and, therefore, alteration of neuronal gene expression [12]. CUL4B catalyzes histone ubiquitination (H2AK119) and facilitates the recruitment of polycomb repressive complex 2 (PRC2) to repress the expression of various tumor-suppressor genes, most importantly PTEN and p16 [13].

Here, we employed an siRNA approach to explore the role of CUL4B in cell lines and primary PM cells. We demonstrated that CUL4B knockdown resulted in cell death and reduced colony formation. Although various cellular effects are similar to CUL4A knockdown, we identified the unique effect of CUL4B knockdown in PM being the regulation of TGFβ1 and one of TGFβ-responsive genes, MMP2, expression. Overall, our data suggest that high expression of CUL4B may drive PM aggressiveness via regulation of TGFβ1 expression.

## 2. Results

### 2.1. Reduced Colony Formation of PM Cell Lines and Primary Cells after CUL4B Knockdown

**Figure 1:**
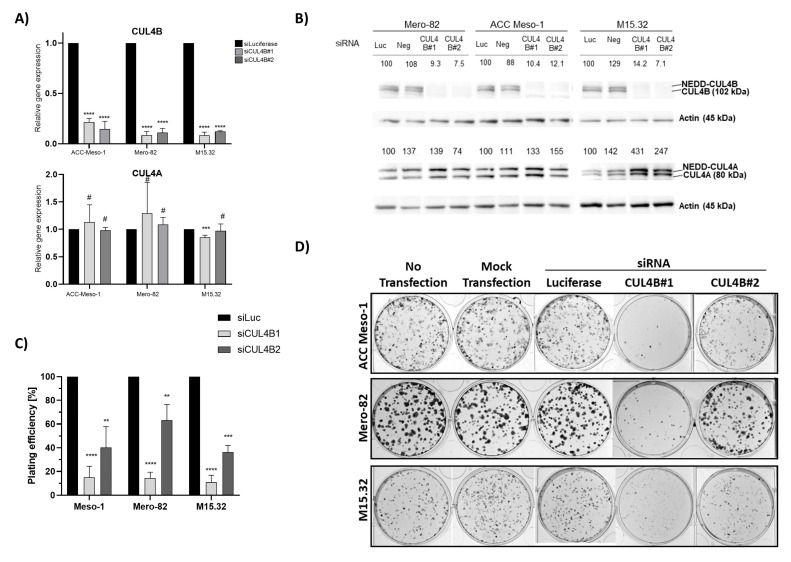
(**A**) RT-qPCR and (**B**) Western blot showing knockdown efficiency of CUL4B siRNAs in the 3 cell lines using 2 different siRNA sequences, at 72 h after transfection. NEDD-CUL4B, neddylated CUL4B. Numbers indicate the relative expression of the proteins (normalized with Actin and compared to non-silencing control (siLuc). (**C**) Colony-formation assay showing reduced plating efficiency of cells following CUL4B knockdown compared to non-silencing control (siLuciferase, siLuc). (**D**) Representative images of CFA data, plated with 500 cells. All the graphs show the mean ± SD from at least two independent experiments. ** *p* < 0.01, *** *p* < 0.001, **** *p* < 0.0001, # not significant.

We employed two different siRNAs (CUL4B#1 and CUL4B#2) to knock down CUL4B in two PM established cell lines (ACC Meso-1, Mero-82) and one PM primary cell culture (M15.32) (Supplementary Figure S1). Both siRNAs showed efficient downregulation of >80% of CUL4B mRNA (Figure 1A). At least an 85% efficiency of protein knockdown was achieved in all cell lines (Figure 1B) (maximum remaining protein expression was 14.2% for M15.32 with CUL4B#1). We initially employed two different non-silencing controls (siLuciferase and siNeg), but siNeg affected the viability of all PM cells, so this control was omitted from further experiments. We showed that both siRNAs are specific for CUL4B without downregulating the expression of its paralog “CUL4A” (Figure 1A). However, upregulation of CUL4A protein was observed following CUL4B depletion (Figure 1B). Likewise, siCUL4A effectively knocked down CUL4A without changing the CUL4B gene’s expression (Supplementary Figure S2A), but also resulted in an increased CUL4B protein level (Supplementary Figure S2B). 

We observed reduced colony formation following CUL4B knockdown (Figure 1C,D) in all cell lines. A stronger effect was observed with CUL4B#1 siRNA. This may be due to the likeliness of the higher knockdown efficiency of CUL4B#1 as reflected by the higher CUL4A protein stabilization, although differences in CUL4B protein expression were not seen (likely due to the limitation of the assay). 

### 2.2. Downregulation of CUL4B Induced Cell Death and Delayed Cell Proliferation, but Did Not Change Cell Sensitivity to Cisplatin

**Figure 2:**
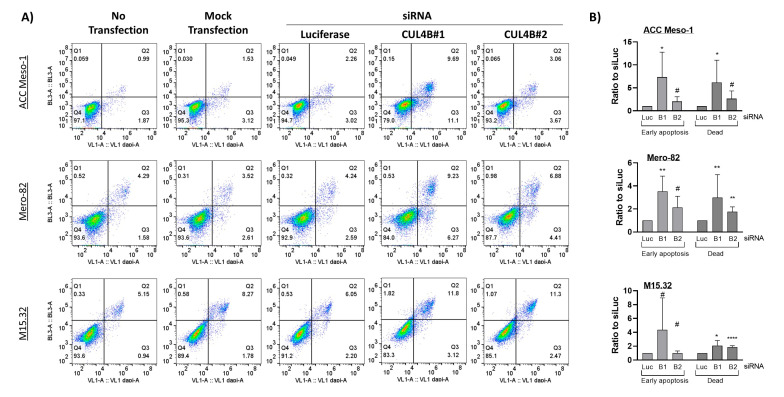
(**A**) Flow cytometric analysis of apoptosis and cell death using Annexin V (x-axis) and Zombie NIR (y-axis) staining. The upper right quadrant (Q2, Zombie+/Annexin V+) represents the dead cell population; the lower right quadrant (Q3, Zombie−/Annexin V+) represents the early apoptotic cell population. (**B**) Summary of cell death and apoptosis induced by CUL4B knockdown in the three cell lines. Due to the high variation of the baseline early and apoptotic cell numbers between each experiment, the number of positive cells was normalized to the control (siLuc) from the same experiment. All the graphs show the mean ± SD from at least three independent experiments. * *p* < 0.05, ** *p* < 0.01, **** *p* < 0.0001, # not significant.

**Figure 3:**
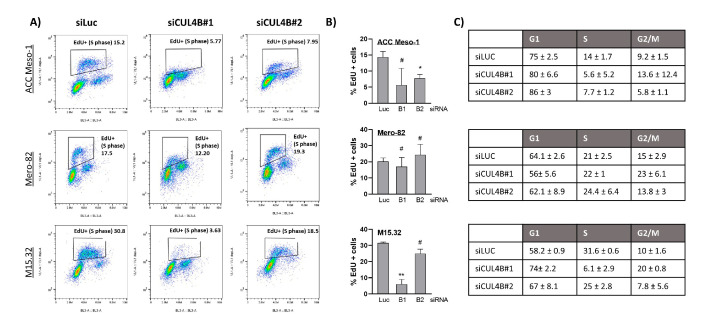
(**A**) EdU incorporation assay of cells following CUL4B knockdown. Cells were treated with EdU for 2 h prior to staining for EdU (y-axis) and DNA (PI; x-axis) followed by flow cytometric analysis. EdU-positive cells represent cells undergoing DNA synthesis (S-phase; EdU+ population). (**B**) Summary of EdU-positive cells detected following CUL4B knockdown from at least 2 independent experiments. (**C**) Summary of cell cycle distribution (mean ± SD) from the EdU assay. * *p* < 0.05, ** *p* < 0.01, # not significant.

Staining with a cell death marker (Zombie NIR) and apoptotic marker (Annexin V) further revealed slightly increased numbers of early apoptotic cells (Q3) and, more significantly, dead cells (Q2) (Figure 2) after CUL4B knockdown. EdU incorporation assays further suggested reduced numbers of cells entering the S-phase following CUL4B knockdown in ACC Meso-1 and M15.32 (Figure 3A,B), but the data did not reach statistical significance. The reason was the high variation of EdU-positive cells between each replicate (in both the control and CUL4B knockdown samples). There was no alteration of EdU-positive cells in Mero-82 after knockdown. Analysis of the cell cycle revealed cell cycle arrest in the G1-phase for all cell lines, except Mero-82. CUL4B has been shown to be involved in nucleotide excision repair following DNA damage; we, thus, employed ACC Meso-1 to test whether CUL4B downregulation changes cell sensitivity to cisplatin, the standard of care chemotherapeutic agent for PM. We did not detect any change in cell sensitivity to cisplatin following CUL4B knockdown compared to the non-silencing control (Supplementary Figure S3).

### 2.3. Reduced Expression of YAP1 and Its Target Genes Following CUL4B and CUL4A Knockdown

**Figure 4:**
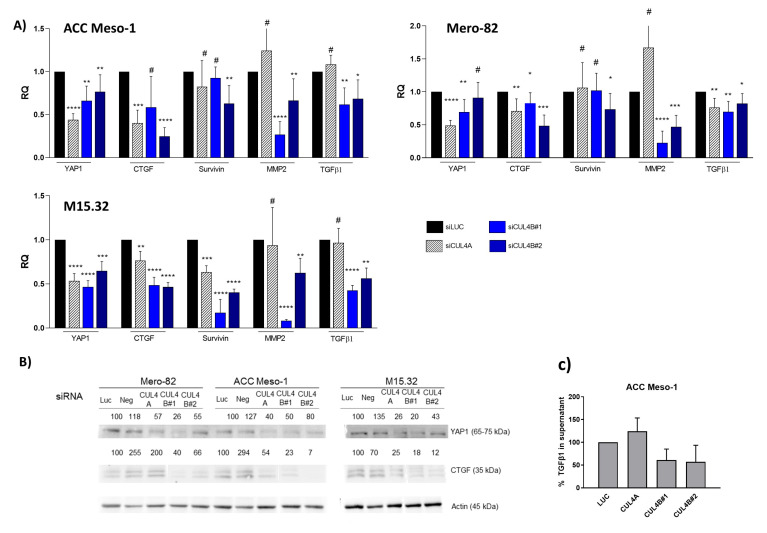
(**A**) RT-qPCR data showing changes in gene expression after CUL4A and CUL4B knockdown. (**B**) Western blot confirms changes of YAP and CTGF in protein level. Numbers indicate the relative expression of the proteins (normalized with Actin and compared to the non-silencing control (siLuc). (**C**) ELISA of total TGF-β1 in the cell culture supernatant of ACC Meso-1 after 48 h of siRNA transfection. ELISA was performed in 2 independent experiments, and the difference was not statically significant due to the high standard variation. * *p* < 0.05, ** *p* < 0.01, *** *p* < 0.001, **** *p* < 0.0001, # not significant.

It has been shown that theCUL4 complex, known as the CRL4 complex, regulates the tumor suppressor Hippo pathway. The CRL4 complex ubiquitinated and inhibited Lats1 and 2, thereby promoting the activation of the transcriptional coactivator, YAP1, in the nucleus. Active YAP1 then supported tumorigenesis by inducing transcription of its target genes [14]. To identify whether this effect is one of the mechanisms driving cell growth arrest, we analyzed the level of YAP1 target genes, CTGF and survivin, following CUL4A and CUL4B knockdown. Significant downregulation of YAP1 and CTGF was detected after knockdown of CUL4A and CUL4B (Figure 4A,B). Nevertheless, the possibility that the change in YAP1 and CTGF expression was a result of cell cycle alterations cannot be ruled out, as CUL4A knockdown also resulted in cell cycle arrest in the G1-phase (Supplementary Figure S2C,D). Survivin was downregulated significantly only in primary cells, M15.32.

### 2.4. Distinct Function of CUL4B in PM in the Regulation of Transforming Growth Factor Pathway

YAP1 expression was reduced following knockdown of both CUL4 paralogs. However, the downregulation of CTGF expression was more pronounced after CUL4B knockdown on both the gene expression and protein levels (Figure 4A,B). We, thus, hypothesized that CTGF is regulated by CUL4B through an additional pathway. It has been demonstrated that CTGF expression was regulated by TGF-β signaling, upstream of YAP1 in mesothelioma [15]. This prompted us to investigate whether CUL4B knockdown affects TGF-β signaling. We tested the expression of MMP2, a TGF-β1 target gene, in PM [15]. Indeed, MMP2 was downregulated following CUL4B knockdown, but showed minimal or no change following CUL4A knockdown (Figure 4A). Furthermore, TGF-β1 expression itself was reduced after CUL4B knockdown (Figure 4A). Nevertheless, the expression levels of MMP2 and TGFβ do not always correlate. This might be due to different alterations in each cell line as MMP2 expression can also be transcriptionally regulated by other factors such as NF-κB [16]. ELISA detection of total TGF-β1 in the cell culture supernatant of ACC Meso-1 showed that, indeed, the TGF-β1 levels were reduced following CUL4B depletion (Figure 4C). The levels of TGF-β1 in M15.32 were below the detection limit, even after 72 h of incubation. The number of dead cells of siRNA-treated Mero82 were above 50%, and therefore, it was inconclusive whether TGF-β1 detected in the supernatant was also a result of protein released from dead cells. Therefore, the data from ELISA are only valid for ACC Meso-1. The average cell numbers at harvest for ACC Meso-1 (as a percentage of siLUC) were 80%, 71%, and 90% for siCUL4A, CUL4B1, and CUL4B2 after 48 h of transfection, respectively.

### 2.5. TGF-β1 Treatment Did Not Rescue Tumor Cell Growth

**Figure 5:**
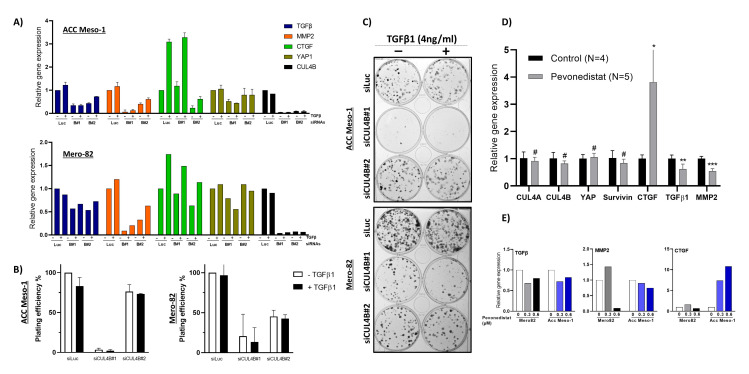
(**A**) RT-qPCR showing changes in gene expression after the treatment of cells with TGF-β1 (4 ng/mL) for 6 h. (**B**) Colony-formation assay showing no difference in plating efficiency of the cells treated with TGF-β1 (4ng/mL). (**C**) Representative CFA images. (**D**) Analysis of gene expression in tissue from the ACC Meso-1 xenograft treated with pevonedistat from our previous publication [6]. (**E**) RT-qPCR showing the change in the gene expression of TGF-β1, MMP2, and CTGF in cells treated with pevonedistat in vitro. Data are presented as the mean ± SD. Where no SD is present, the experiment was performed only once with the qPCR technical replicate. * *p* < 0.05, ** *p* < 0.01, *** *p* < 0.001, # not significant.

We further tested whether reduced cell growth observed after CUL4B knockdown can be rescued by exogenous TGF-β1 treatment. We, thus, treated the cells with siRNA and performed the colony-formation assay in the presence and absence of exogenous TGF-β1, using the concentration (4 ng/mL) previously described to stimulate the growth of non-malignant mesothelial cells in 3D culture [15]. Treatment with TGF-β1 stimulated the expression of TGF-β1, MMP2, and CTGF, showing the activity of TGFβ1 in our experiment condition (Figure 5A). Nevertheless, the treatment failed to rescue colony formation following CUL4B knockdown (Figure 5B,C). TGF-β1 treatment also did not stimulate colony formation in the control condition (siLUC) (Figure 5B,C). These data indicate that this unique effect of CUL4B via the TGF-β1 pathway does not regulate the tumor growth itself in our culture condition. Due to the well-known functions of TGF-β1 in the regulation of the tumor microenvironment, it is likely that CUL4B regulates the tumor microenvironment through TGF-β1 regulation.

### 2.6. Treatment with NEDD Inhibitor Resulted in Downregulation of TGF-β1 In Vitro and In Vivo 

In our previous study, we investigated the effect of cullin inhibition by the inhibitor of protein neddylation, pevonedistat, in vitro and in vivo. Protein neddylation is essential for post-translational modification of all cullins [7] including CUL4B; thus, pevonedistat is a rather broad-spectrum inhibitor. In vitro, ACC-Meso1 cell lines were resistant to pevonedistat and did not show any cell death even when treated with a high concentration. However, the pevonedistat-treated ACC Meso-1 xenograft showed increased cell death compared to the control, suggesting that the treatment might have effects on tumors via the tumor microenvironment. Nevertheless, it is worth mentioning that the change of drug sensitivity can also be modulated by other factors such as hypoxia and extracellular matrix components, which were absent in the in vitro condition. From the evaluation of the cells associated with the tumor microenvironment, we observed significantly reduced numbers of macrophages in ACC-Meso1-treated tumors [6]. It is well known that macrophages play an essential role in the tumor microenvironment and are mediated by TGF-β. We, thus, hypothesized that the reduced macrophage recruitment observed in vivo is mediated by reduced TGF-β signaling by pevonedistat. We performed RT-qPCR analysis of the tumor tissues collected from the xenograft from ACC Meso-1 using human-specific PCR primers. Indeed, we observed significant downregulation of TGF-β1 and MMP2 in the tumor tissues treated with pevonedistat (Figure 5D). In vitro treatment of cells with pevonedistat also resulted in TGF-β1 and MMP2 downregulation (Figure 5E). However, CTGF was highly upregulated, most likely through a different mechanism due to a pleiotropic effect of pevonedistat, especially by inducing tissue fibrosis.

### 2.7. Effects of Combined CUL4A and CUL4B Knockdown and Inhibition of Protein Degradation by Proteasome Inhibitor 

**Figure 6:**
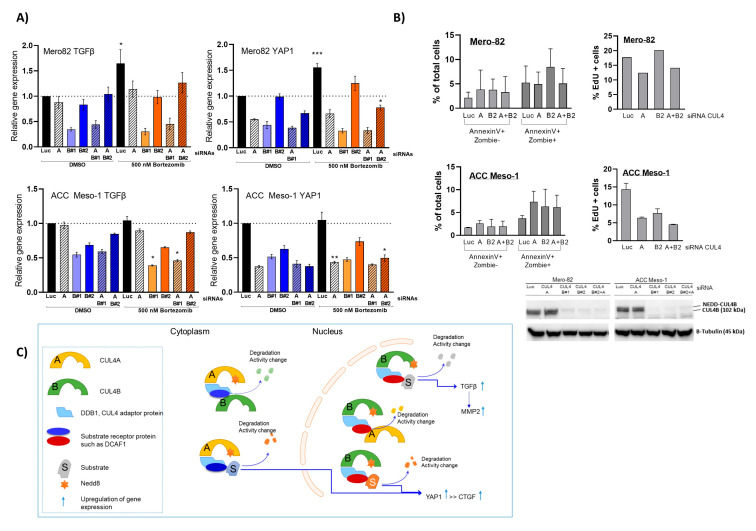
(**A**) RT-qPCR showing changes in gene expression after treatment of cells with siRNAs, single- and double-knockdown for 48 h, followed by treatment with the DMSO control or 500 nM Bortezomib for an additional 6 h. The data are presented as the mean ± SD from at least 2 independent experiments. * *p* < 0.05, ** *p* < 0.01, *** *p* < 0.001 statistically not significant where no symbol is present. (**B**) Assay of cell death and apoptosis by Annexin V and the EdU incorporation assay staining following single- and double-knockdowns with CUL4A and CUL4B#2. Western blot shows the efficiency of CUL4B knockdown. (**C**) Illustration of our hypothesis regarding the role of CUL4B and CUL4A in PM. We show here a simplified model of the CUL4 complex comprising neddylated CUL4A or CUL4B with the adaptor protein (DDB1) and a substrate receptor protein. CUL4B is mainly localized in the nucleus, whereas CUL4A is mainly in the cytoplasm. This leads to the hypothesis that they engage different substrate receptor proteins to regulate the expression level of YAP1 and CTGF, via the regulation of a so-far unknown factor. Independent of CUL4A, CUL4B regulates the expression of TGFβ1 by a so-far unresolved complex and mechanisms. It is likely that the paralogs regulate the stability of each other likely by the ubiquitination process.

We next performed the knockdown of both CUL4A and CUL4B simultaneously. The knockdown by cotransfection with CUL4A and CUL4B siRNAs showed less efficiency compared to the single knockdown (Supplementary Figure S4), but overall still reached ≥90% for CUL4B and ≥60% for CUL4A in the combined knockdown. Co-knockdown of CUL4A and CUL4B did not further increase the effect on the downregulation of TGFβ1 and the YAP1 gene’s expression (Figure 6A). We performed the EdU incorporation assay and Annexin V staining using combined knockdown with CUL4A and CUL4B2. We also observed no further increase in the effect on cells by both assays with double-knockdown compared to the single-knockdown (Figure 6B). Next, we performed treatment with the proteasome inhibitor Bortezomib to evaluate whether the regulation of gene expression was mediated by ubiquitin proteasome degradation. In contrast to cullin inhibition, we observed significantly increased expression of TGFβ1 and YAP1 with Bortezomib treatment in Mero82. We detected partially rescued expression of both genes in CUL4A and CUL4B knockdown conditions (Figure 6A). For ACC-Meso1 cells, we did not detect significant changes during this treatment period. 

## 3. Discussion

Previously observed correlations between CUL4B levels and clinical outcomes suggested that CUL4B likely plays a role in PM progression [6,17], but the mechanism has been so far unexplored. Here, we employed PM cell culture and primary cells to demonstrate the mechanism of CUL4B in the regulation of PM progression. We showed that CUL4B is important for tumor cell growth, similar to its paralog, CUL4A. In addition, we demonstrated that CUL4B regulates TGF-β1 signaling in PM cells and this may affect the tumor microenvironment in part by signaling macrophage recruitment. Our data further confirmed the important role of CUL4B in cancer and support the oncogenic role of CUL4B in PM.

Similar to its paralog, CUL4B knockdown resulted in immediate alteration of the cell cycle and cell viability. This aspect of CUL4A and CUL4B in the regulation of cell survival is well known, mainly due to their shared functions in the degradation of crucial cell cycle regulators including p21, p27, and CDT1 [6,7,11]. Nevertheless, in terms of cell cycle arrest, each cell line seemed to respond differently. This is most likely due to the different growth rate and the proportion of cells in each cell cycle phase during the treatment. In addition, this might depend on other cell cycle regulators that are individually altered in each cell line. Knockdown of both paralogs at the same time did not add up to the effects of the single-knockdown, suggesting that CUL4A and CUL4B employ different mechanisms to regulate PM cell growth and survival. This is most likely explained by the functional complexity, as CUL4A and CUL4B form a complex with DDB1 that links CUL4 to its substrate receptor protein [18]. Thus far, there have been over 50 putative substrate receptors identified for CUL4 ubiquitin ligase that possess different biological functions [19]. The increased protein expression of CUL4B following CUL4A knockdown and vice versa suggest that both paralogs might induce the degradation of each other. Nevertheless, this has to be further investigated in more detail.

The novelty of our data is the unique effect of CUL4B in the regulation of TGFβ signaling in PM, which likely plays an important role in the regulation of the tumor microenvironment. Although the two paralogs share various similar functions in cells, we found that the expression of CUL4B correlated with PM disease outcomes, while CUL4A expression did not show any tendency [6]. Thus, the unique action of CUL4B in the regulation of TGFβ signaling might represent an important aspect of CUL4B in the progression of PM. Our data are also supported by a recent study by Liu, L et al. that employed publicly available datasets to identify correlations between CUL4B and the PM tumor microenvironment. The study employed gene expression data from the TCGA and GEO databases to demonstrate significant correlations between CUL4B expression and clinical outcomes and immune cell infiltration [20]. Confirming our previous data, the analyses showed that CUL4B expression was elevated in PM tumor tissue compared to normal pleura, and high CUL4B expression was associated with short the progression-free survival of PM patient cohorts. They further divided PM patients into two groups according to CUL4B expression and showed the enrichment of the TGF-β signaling pathway in the CUL4B-high compared to the CUL4B-low group. CUL4B expression was associated, both positively and negatively, with an abundance of some immune cells such as T-cell subpopulations and dendritic cells. Altogether, gene expression data from the publicly available databases support our hypothesis regarding the role of CUL4B in the regulation of TGF-β signaling, as well as the tumor microenvironment in PM tumors.

TGF-β is an influential cytokine ubiquitously expressed and secreted by both inflammatory and cancer cells [21]. There are three forms, of which TGF-β1 is the most-relevant [22]. TGF-β can promote or suppress tumor growth depending on the cancer stage and cancer–microenvironment interactions [23]. It has been long known that TGF-β plays an important role in PM biology. Gerwin et al. found that PM cells secrete both the active and inactive form of TGF-β [24]. Furthermore, TGF-β is more prevalent in pleural effusions of patients with PM compared to patients with breast cancer or non-small cell lung cancer [25]. TGF-β, furthermore, has a crucial role in epithelial-to-mesenchymal transition (EMT) [26]. PM cells can undergo EMT when stimulated by TGF-β [27]. Fassina et al. were able to show that tumors of the most-aggressive sarcomatoid subtype showed higher expression of EMT transcriptional regulators (e.g., SNAIL, SLUG, TWIST) and higher expression of mesenchymal markers (such as vimentin and MMP9) compared with tumors of the biphasic and epithelioid subtypes [28], which further confirms the importance of TGF-β in PM tumor growth and invasion. Although TGF-β did not stimulate tumor cell growth (determined by 2D colony-formation assay) in our study, we noticed a change in the shape of colonies (Figure 5C). TGF-β treated colonies were more diffuse compared to untreated ones. This might indicate increased motility related to EMT; nevertheless, this phenomenon cannot be concluded by our assays. 

An immunosuppressive role (e.g., by inhibiting NK cells, weakening cytotoxic lymphocytes) of TGF-β has been demonstrated [29] previously. TGF-β also has an important role with respect to tumor associated macrophages (TAMs), but the exact mechanism of regulation remains elusive [30]. It has been shown that TGF-β secreted from tumor cells (e.g., in oral squamous cell carcinoma) induced the polarization of macrophages to the M2 tumor-promoting type [31,32]. The relationship between TGF-β1 signaling and tumor-associated macrophages has also been recently demonstrated in PM. Gene expression analysis in PM demonstrated that a high expression of TGF-β1 and MMP2 was associated with the presence of tumor-promoting M2 macrophages. Patients with high TGF-β1 expression and MMP2 showed worse survival [33]. Another and the most-recent finding using a high-dimensional transcriptomic approach further identified a strong positive correlation between TGF-β1 and tumor-associated macrophage genes, as well as M2 macrophages in the PM biphasic subtype [34]. Altogether, these data strengthen the relationship between CUL4B, TGF-β1 signaling, and the regulation of macrophages in PM progression. 

A better understanding of the mechanisms through which CUL4B regulates TGF-β signaling in PM will better elucidate its oncogenic role in cancer. Given the known role of CUL4B in transcriptional regulation, the regulation of TGF-β signaling is likely through this mechanism. This hypothesis is supported by a recent study in breast cancer showing that CUL4B interacts with several HDAC-containing complexes and regulates EMT also in part via TGF-β regulation [35]. An increased CUL4B protein level after CUL4A knockdown did not compensate for the downregulation of TGF-β and YAP expression. Both CUL4A and CUL4B regulate protein ubiquitination and degradation by forming a complex with DDB1 and substrate-specific adaptors. The substrate-specific adaptor is the key molecule that regulates substrate specificity [19]. We hypothesized that, in the CUL4A knockdown condition, although CUL4B protein was increased, the gene expression could not be rescued due to the limited amount of substrate-specific adaptor. 

Furthermore, it is important to identify whether CUL4B upregulation regulates other cells and immune cells in the tumor microenvironment. Immunotherapy was approved by the FDA for patients with unresectable PM and is being tested in several clinical trials; nevertheless, not all patients benefitted from the treatment [36]. Thus, understanding additional players in the regulation of the tumor immune microenvironment may help to identify markers or additional targets that can be exploited to improve the treatment efficacy. Pevonedistat, a protein neddylation inhibitor also targeting cullins, is currently being tested in PM in a clinical trial, NCT03319537. Our work provides additional data for the better understanding of the mechanism of the clinical response to pevonedistat and its treatment efficacy in PM.

## 4. Materials and Methods

### 4.1. Cell Lines and Primary Cells

The malignant cell lines Mero-82 (The European Collection of Cell Cultures (ECACC), Salisbury, UK) and ACC-Meso-1 (RIKEN Bioresource, Saitama, Japan) were acquired from the indicated providers. M15.32 is a primary cell line cultured from pleural effusion from a patient with PM treated at the University Hospital Zurich. The patient signed an informed consent, and this study was approved by the cantonal ethical committee of Canton Zürich (BASEC-No. 2020-02566). Primary cells were cultured in an RPMI medium ATCC modification (Gibco^TM^, A1049101, Life Technology Europe BV, Zug, Switzerland) containing 2 mM glutamine (Sigma-Aldrich, Buchs, Switzerland), 10% fetal bovine serum (FBS) (Biowest, Nuaillé, France), penicillin/streptomycin (Biowest, Nuaillé, France), heparin 2 µg/mL (SigmaAldrich, Buchs, Switzerland), hydrocortisone 2 µg/mL (SigmaAldrich, Buchs, Switzerland), and human epidermal growth factor (20 ng/mL, hEGF) (Peprotech, London, UK) and maintained at 37 °C and 5% CO_2._ The identification of PM was achieved using immunohistochemical staining of epithelial and PM markers including pan-Cytokeratin, podoplanin, calretinin, and BAP1 staining. M15.32 showed nuclear BAP1 loss, identical to that of the original tumor (Supplementary Figure S1A) and no BAP1 detectable by Western blot (Supplementary Figure S1B). For the experiments, all cells were cultured in an RPMI medium ATCC modification (Gibco^TM^, A1049101, Life Technology Europe BV, Zug, Switzerland) containing 2 mM glutamine (Sigma-Aldrich, Buchs, Switzerland) and 10% fetal bovine serum (FBS) (Biowest, Nuaillé, France) at 37 °C and 5% CO_2_. To avoid superposed growth, the cells were regularly detached and passaged using a mild enzymatic solution (trypsin-EDTA, Biowest, Nuaillé, France). All cell lines were regularly tested for the absence of mycoplasmas.

### 4.2. siRNA Transfection

Forward transfection was performed with 20 nM siRNA using Lipofectamine^®^ RNAiMAX (Invitrogen 13778150, Life Technology Europe BV, Zug, Switzerland). The siRNA sequences are provided in Supplementary Table S1. Cells were seeded in a 6-well plate (ACC Meso-1 30,000 cells/well, Mero82 and M15.32 40,000 cells/well) in the growth medium to reach 40% confluency of adherent cells after 24 h. On the next day, the siRNA/Lipofectamine complex mixture was prepared as follows: per well, 5 µL Lipofectamine^®^ was diluted in 250 µL Opti-MEM^®^ medium (Invitrogen, 31985-062 Life Technology Europe BV, Zug, Switzerland) and mixed with 200 nM siRNA duplex suspension in 250 µL Opti-MEM^®^. The transfection complex was allowed to form for 15 min at room temperature. Next, a total of 500 µL of siRNA Lipofectamine^®^ complex mixture was pipetted dropwise onto the cells containing 2 mL growth medium (total volume 2.5 mL) in culture and cultured for 72 h for RNA extraction, Western blotting, and cell cycle analysis.

### 4.3. Double-siRNA Transfection and Treatment with Bortezomib

We performed siRNA forward transfection as described above. For double-transfection, 20 nM of each siRNA was used with 5 µL Lipofectamine^®^ per well. Cells were cultured afterwards for 48 h when Bortezomib (Selleckchem PS-341, S1013, Houston, TX, USA) was added to each well. We added 6.25 µL Bortezomib (200 µM in 25% DMSO/water) to each well containing 2.5 mL culture medium (final concentration 500 nM) medium and incubated for another 6 h prior to RNA extraction. Control wells were treated with an equal amount of 25% DMSO/water.

### 4.4. Colony-Formation Assay

The colony-formation assay was used to determine the ability of a single cell to undergo division and form a colony. At 48 h after siRNA transfection as described above, cells were trypsinized and counted. We seeded different numbers of cells (250, 500, 1000 cells) per well (each in duplicate) of a 6-well plate and cultured in 2 mL of culture medium. On Day 4 after seeding, 500 µL of fresh culture medium was added. On Day 6, 1.5 mL of culture medium was aspirated and replenished with 1.5 mL of fresh culture medium. On day 8, cells were washed with ice-cold Dulbecco’s phosphate-buffered saline (DPBS, Biowest, Nuaillé, France) and fixed in ice-cold absolute methanol for 20 min at −20 °C. Afterwards, 1.5 mL of crystal violet was applied to each well and incubated for a minimum of 1 h. Crystal violet was washed with water, and the colonies were imaged using Fusion FX7 (Witec AG, Sursee, Switzerland). Colony counting was performed manually using the Image J [37] manual cell counting function. From different seeding numbers, we only counted the wells with clearly visible isolated colonies. Finally, we used the average data of plating efficiency (number of colonies/number of cells seeded) of the duplication for further analysis. For the rescue experiment, we prepared a stock solution of 100 µg/mL of recombinant human TGF-β1 (Peprotech 100-21, London, UK) in 10 mM citric acid pH 3.0 and diluted to 1 µg/mL of working stock (250X) in DPBS containing 0.1% BSA. Then, 48 h after transfection, cells were counted and the cell suspension was seeded into 6-well plates in 2 mL of complete culture medium, for the control and TGF-β1-treated. Recombinant human TGF-β1 was added to the culture medium immediately after seeding at the final concentration of 4 ng/mL. The same amount of protein diluent was applied to the no-TGF-β1 control. Medium change was performed on Day 3 after seeding. After aspiration of 1 mL of medium, we replenished with 1 mL of fresh medium without or with 4 ng/mL of TGF-β1. Colonies were fixed and stained on Day 7 after seeding.

### 4.5. Analysis of Apoptosis and Proliferation by Flow Cytometry

Cells were seeded in 6-well plates and transfected with siRNAs as described above. At 72 h after transfection, they were collected for apoptosis and the cell cycle assay.

For apoptosis, we collected the cell culture supernatant (containing floating dead cells), and the adherent cells were detached with trypsin for 5 min. After stopping the trypsin reaction with fresh culture medium, the cell suspension was pooled with supernatant. Cells were washed with 3 mL of DPBS followed by 3 mL of Annexin V binding buffer containing 10 mM HEPES/NaOH pH7.4, 140 mM NaCl, and 2.5 mM CaCl_2_ and pelleted by centrifugation at 400× *g* for 5 min. Afterwards, we resuspended cells in 100 µL of Annexin V binding buffer and added 5 µL of Annexin V (Annexin V Pacific Blue conjugate, Molecular Probe; A35122, Life Technology Europe BV, Zug, Switzerland) and 1 µL of Zombie (Zombie NIR, Biolegend 423105, Amsterdam, The Netherlands). The complex was incubated at room temperature in the dark for 15 min. We then added 400 µL of Annexin V binding buffer to the suspension, kept on ice, filtrated through 0.22 µm filters and immediately analyzed with an Attune cytometer (Applied Biosystems, Life Technology Europe BV, Zug, Switzerland).

For the BrdU incorporation assay using the Click-iT™ EdU Pacific Blue™ Flow Cytometry Assay Kit (Invitrogen C10418), we treated the adherent cells with 10 µM EdU for 2 h in the complete medium. Afterwards, cells were washed and detached using trypsin for 5 min. The cell suspension was pelleted by centrifugation and washed with 3 mL DPBS. We resuspended the cells in 100 µL of 1%BSA in PBS and fixed with 100 µL of Click-iT^®^ fixative by adding dropwise and incubated for 15 min. After fixation, we washed the cells with 3 mL of 1%BSA in PBS and permeabilized the cells with 100 µL of 1X Click-iT^®^ saponin-based permeabilization buffer for 15 min. Afterwards 500 µL of Click-iT^®^ reaction cocktail (prepared according to the manufacturer’s instructions) was added and incubated for another 30 min at room temperature in dark. We pelleted the cells, washed them once with 3 mL of permeabilization buffer, and discarded the supernatant. The cell pellet was resuspended in 500 μL of FxCycle PI/RNase (Invitrogen: F10797), filtrated, and immediately analyzed by the Attune cytometer.

We used an Attune acoustic focusing cytometer for data acquisition (Attune, Applied Biosystems, Life Technology Europe BV, Zug, Switzerland). Data analysis was performed with the FlowJo software v.10 (BD Biosciences, San Jose, CA, USA). We excluded debris and gated single cells for the analysis.

### 4.6. Viability MTT Assay for Drug Sensitivity

We seeded ACC Meso-1 at 2000 cells/100 µL/well in a 96-well plate. Cells were transfected with 20 nM siRNA at 24 h after seeding. We used 0.2 µL of Lipofectamine^®^ diluted in 10 µL of Opti-MEM^®^ medium per well and mixed with 240 nM siRNA duplexes diluted in 10 µL of Opti-MEM^®^. The transfection complex (20 µL of complex containing 120 nM siRNA) was allowed to form for 15 min and was added dropwise to cells containing 100 µL of medium (final 20 nM). At 24 h after transfection, we aspirated the cell culture medium and replaced with complete medium containing an increasing concentration of cisplatin ranging from 2.5–40 µM (stock 1 mg/mL in 0.9% NaCl, Actavis, Regensdorf, Switzerland) using the diluent (0.9% NaCl) as the untreated control. At 48 h after cisplatin treatment, cell survival was measured by a colorimetric assay to rate the metabolic activity of a cell using tetrazole 3-(4,5-dimethylthiazol-2-yl)-2,5-diphenyltetrazolium bromide (MTT) using the protocol previously described [38].

### 4.7. Protein Extraction and Western Blot

After removing the medium and floating cell debris, the adherent cells were enzymatically detached by trypsin, centrifuged, and washed with sterile DPBS. We resuspended the cell pellet in RIPA buffer (50 mM Tris HCl pH 8, 150 mM NaCl, 1% TritonX-100, 0.5% sodium deoxycholate, 0.1% SDS) containing a protease/phosphatase inhibitor cocktail (Cell Signaling, #5872, Danvers, MA, USA) for cell lysis for 20 min followed by pulse sonication 3 times of 10 s (Sonifier^®^ S-450A, Branson Ultrasonics, Urdorf, Switzerland). We then centrifuged the cell extract at 15,000× *g* 4 °C for 20 min and collected the cell lysate. The protein concentration in the lysate was assessed by a Micro BCA Protein Assay Kit (Thermo Fisher, Reinach, Switzerland). Protein denaturation was performed with Laemmli buffer and heated at 95 °C for 10 min. We ran Western blots using homemade Tris-glycine gradient gels (4–20%) in Tris-glycine buffer. The gels were prepared with Rotiphorese^®^ Gel 40 (29:1) (Carl Roth, Karlsruhe, Germany), Tris-HCl/SDS (pH 8.8), water, ammonium peroxodisulfate (APS) (Carl Roth, Karlsruhe, Germany), and tetramethylethylendiamine (TEMED) (Sigma Aldrich, Buchs, Switzerland). The Western blot analysis was conducted using equivalent amounts and volume of protein (range 10–40 µg), which were resolved by sodium dodecyl sulfate-polyacrylamide gel electrophoresis (SDS-PAGE). The proteins were transferred onto a polyvinylidene fluorid membrane (Immuno-Blot PVDF membrane, Bio-Rad, Cressier, Switzerland). Following transfer, the membranes were blocked with 5% blotting grade milk powder (Carl Roth, Karlsruhe, Germany) in Tris-buffered saline containing 0.1% TWEEN-20 (TBST, Sigma Aldrich, Buchs, Switzerland). Primary antibody incubations were carried out over night at 4 °C or for one hour at room temperature. The following primary antibodies were used for Western blot diluted in TBST containing 5% BSA and 0.01% sodium azide: CUL4B (HPA011880, Sigma-Aldrich Buchs, Switzerland, 1:250), CUL4A (2699S Cell Signaling, Danvers, MA, USA, 1:1000), and YAP/TAZ (D24E4 Cell Signaling, Danvers, MA, USA, 1:1000). CTGF (clone L-20 1:500), Actin (clone I-19 1-200), and secondary antibodies including mouse anti-rabbit IgG HRP (sc-2357 1:10,000), mouse anti-goat IgG HRP (sc-2354 1:10,000), and m-IgGκ BP-HRP (sc-516102 1:10,000) were from Santa Cruz, Heidelberg, Germany. The secondary horseradish peroxidase (HRP) antibodies were applied at room temperature at a 1:10,000 dilution in 1% milk in TBST for one hour. Subsequent and in between washes were carried out in TBST. Following the washes, the antigen/antibody complexes were detected by chemiluminescent detection (Clarity Western ECL Western blot substrate, Biorad, Cressier, Switzerland) using a chemiluminescent appliance (Fusion-FX7.826, program FusionCapt). The signal intensity of the bands was also quantified with the FusionCapt program.

### 4.8. RNA Extraction and Reverse Transcriptase Quantitative Real-Time PCR

For RNA isolation, cells were washed with 2 mL DPBS after removal of the culture medium. Immediately after DPBS removal, 1 mL of TRIzol reagent (Invitrogen 15596018, Life Technology Europe BV, Zug, Switzerland) was added for cell lysis. The cell lysate in TRIzol reagent was processed for RNA isolation according to the protocol suggested by the manufacturer. Then, 200–500 ng of RNA was used for cDNA synthesis using PrimeScript RT with gDNA Eraser (TAKARA Bio, S-86901-06-01, Saint-Germain-en-Laye, France). For quantitative real-time PCR, the KAPA SYBR FAST qPCR master mix with ROX (Kapa Biosystems, KK4621 Merck, Buchs, Switzerland) was used and run on a 7500 fast real-time PCR system (Life technology, Life Technology Europe BV, Zug, Switzerland). Gene expression was quantified using 2^-delta delta Ct^ using beta-Actin as the reference gene and compared to the non-silencing control (siLuciferase). The sequences of the used PCR primers are provided in Supplementary Table S2. The primers were used in the PCR reaction at a final concentration of 0.2 µM.

### 4.9. ELISA

ELISA was performed using the cell culture supernatant collected at 48 h after siRNA transfection to minimize variation due to the increased number of dead cells in the siRNA-treated samples. We collected the cell culture supernatant and centrifuged at 400× *g* for 5 min at 4 °C to remove the floating cells. We harvested the supernatant and centrifuged again at 10,000× *g* for 10 min at 4 °C to remove the debris. The supernatant was frozen at −80 °C until the analysis. TGF-β1 ELISA with the activation of latent TGF-β1 was performed using Human TGF-β1 DuoSet ELISA (R&D Systems DY240-05) with DuoSet ELISA Ancillary Reagent Kit 1 (R&D Systems DY007) according to the protocol recommended by the manufacturer. We subtracted the TGF-β1 level with the level of medium from no-cell control wells, treated with mock transfection. Then, the level of TGF-β1 was corrected with the number of living cells and normalized to siLuc.

### 4.10. Statistical Analyses

We used GraphPad Prism v.8.0.0 (San Diego, CA, USA) for testing the differences of the means with parametric *t*-tests.

## Data Availability

Not applicable.

## Supplementary Materials

The following Supporting Information can be downloaded at: https://www.mdpi.com/article/10.3390/ijms241713410/s1.
