# Supplementary material for: Cullin 4B Ubiquitin Ligase Is Important for Cell Survival and Regulates TGF-β1 Expression in Pleural Mesothelioma
Source: Int J Mol Sci. 2023 Aug 29;24(17):13410. doi: 10.3390/ijms241713410 (PMC10487616; doi:10.3390/ijms241713410)
Supplement: Supplementary file 1 [file ijms-24-13410-s001.zip › ijms-2573508-supplementary.pdf]

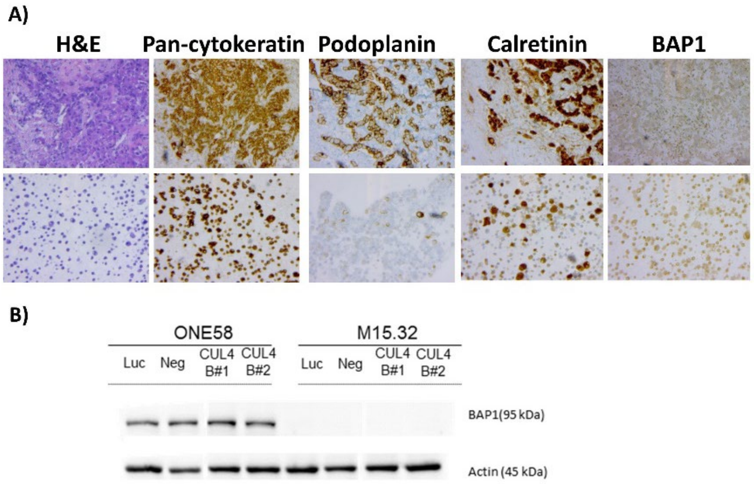

Supplementary figure S1 A) Immunohistochemical staining of PM markers in primary cells cultivated from pleural effusion of a PM patient (M15.32, lower panel) compared to the tumor tissue (upper panel). B) Western blot analysis showing BAP1 loss in M15.32. ONE58 was used as a positive control for BAP1 staining.

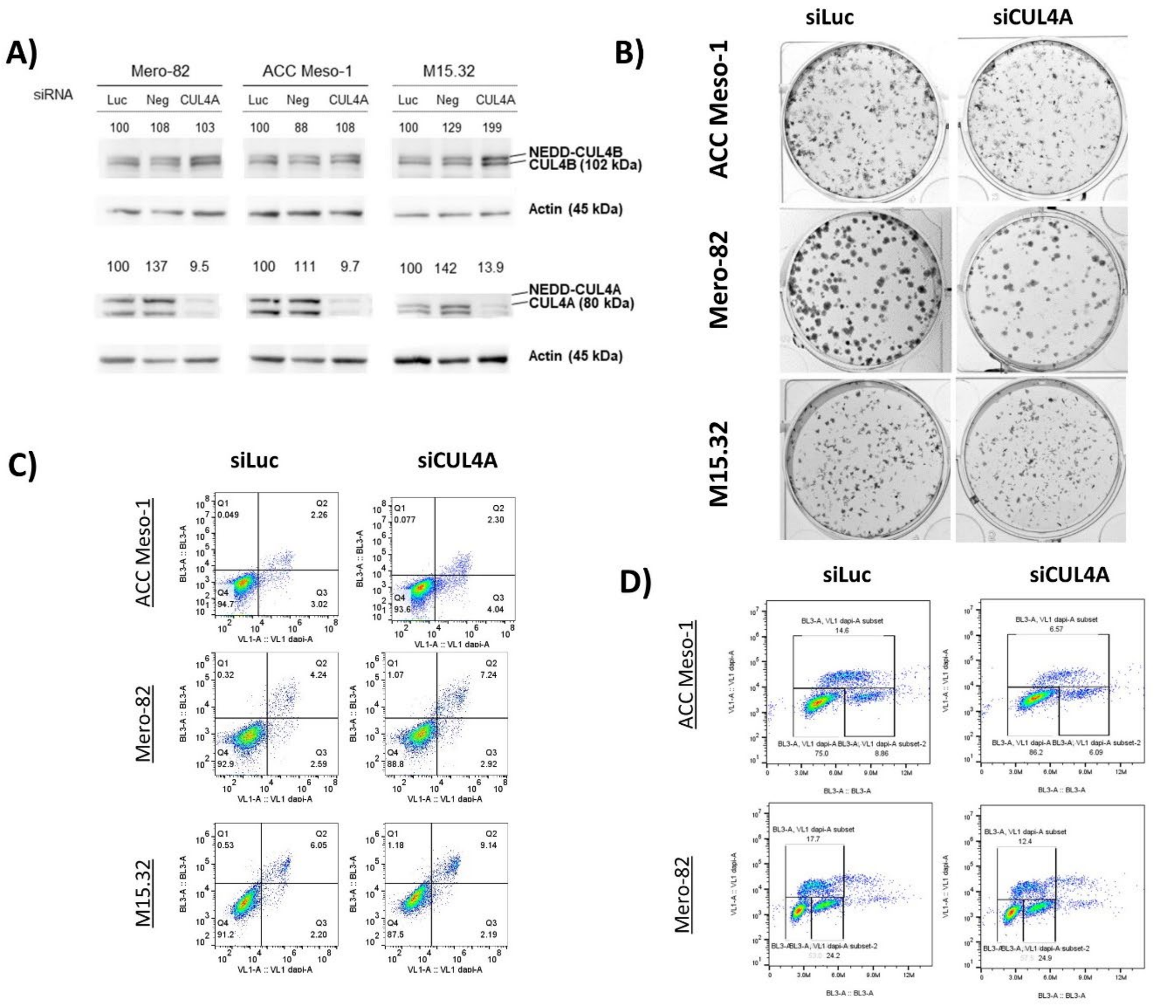

Supplementary figure 2 A) Efficacy of siCUL4A analyzed by western blot. CUL4B protein level was upregulated following CUL4A knockdown. **B)** Reduced colony formation of the cell lines after CUL4A knockdown. Flow cytometry showing increased cell death and apoptosis **(C)** and reduced cell proliferation (S phase) by EdU incorporation assay.

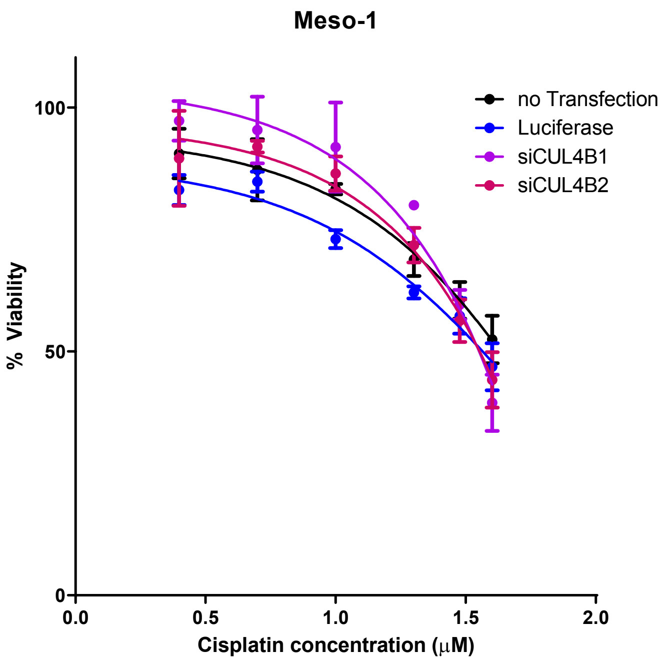

Supplementary figure 3 Response of ACC Meso-1 to increasing concentration of cisplatin determined by viability MTT assay at 48 hours after drug exposure.

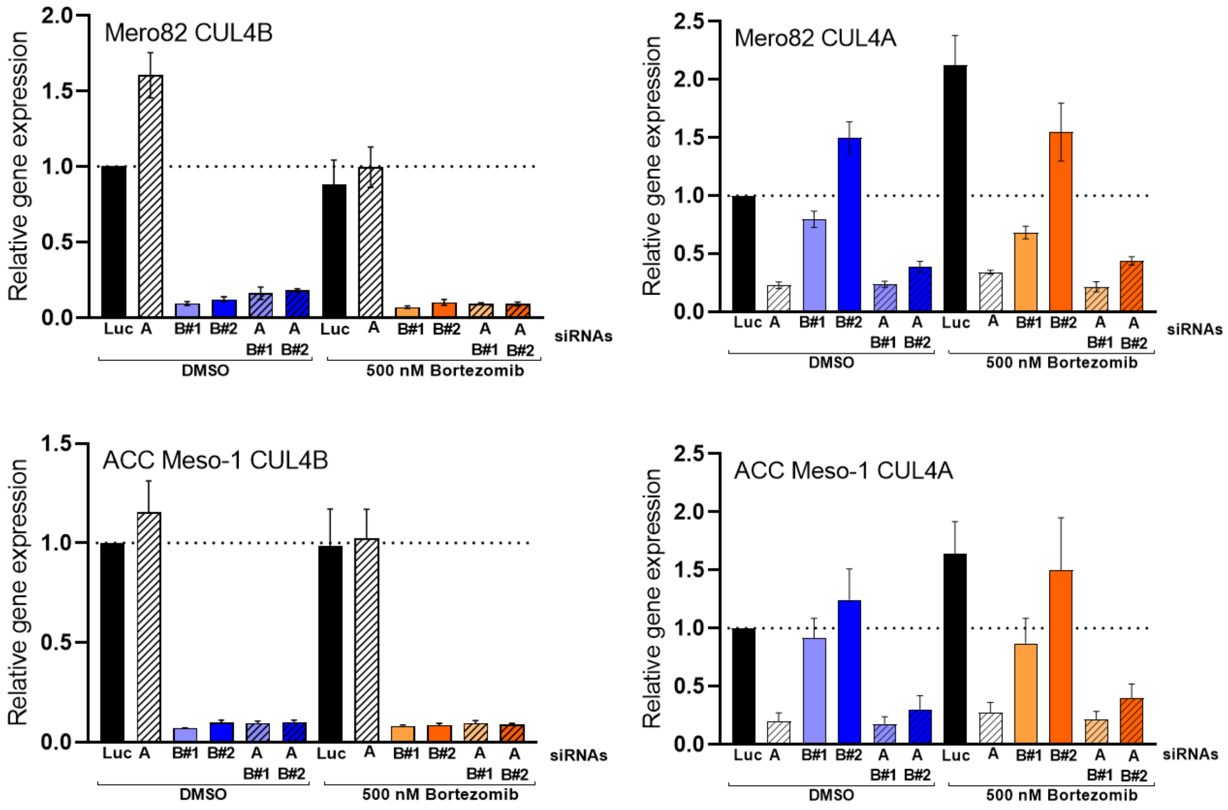

Supplementary figure 4) RT-qPCR showing knockdown efficiency of CUL4A and CUL4B with siRNAs, single and double knockdown for 48 hours, followed by treatment with DMSO control or 500 nM Bortezomib for additional 6 hours.

Table 1: siRNAs sequences and references

Name	Gene	Sequence Sense (5`-3`)	Modification Sense	Modification Antisense	References
Non-targeting control	Luciferase	CGUACGCGGAAUACUUCGATT	dTdT	dTdT	Microsynth; Pre synthesized control siRNAs
CUL4A	CUL4A	GAACCCAUAUUAUU AGUGAUU	rUrU	rUrU	Lovejoy, C.A., et al., DDB1 maintains genome integrity through regulation of Cdt1. Mol Cell Biol, 2006. 26(21): p. 7977-90.
CUL4B_1	CUL4B	AAGCCUAAAUUACCAGAAA	TT overhange	TT overhange	Nakagawa, T. and Y. Xiong, X-linked mental retardation gene CUL4B targets ubiquitylation of H3K4 methyltransferase component WDR5 and regulates neuronal gene expression. Mol Cell, 2011. 43(3): p. 381-91.
CUL4B_2	CUL4B	UAA ACC AGC UGA ACU UAU A	dTdT	dTdT	Designed by Microsynth
Non-targeting negative control	N/A	AGGUAGUGUAAUCGCCUUGtt	dTdT	dTdT	Microsynth; Pre synthesized control siRNAs

Table 2: Sequences for RT-qPCR primers

Gene	Sequence 5′-3′ FW	Sequence 5′-3′ RE
B-Actin	GGACCTGACTGACTACCTCAT	CGTAGCACAGCTTCTCCTTAAT
B-Actin human specific	CCTCGCCTTTGCCGATCC	CGCGGCGATATCATCATCC
CUL4B	GGACATGGGACTGGAGTTATTT	ACCATTCCTTTCCCTCTCAATC
CUL4A	CAGGCACAGATCCTTCCGTT	TCCTGCCAGCACGTGTTAAT
YAP	CAGACAGTGGACTAAGCATGAG	CAGGGTGCTTTGGTTGATAGTA
CTGF	GCCCAGACCCAACTATGATTAG	GGAGGCGTTGTCATTGGTAA
Survivin	GCACCACTTCCAGGGTTTAT	AGGAGCACAGTTGAAACATCTA
